# Growth performance of neonatal calves fed milk replacer 2 vs. 3 times per day

**DOI:** 10.1093/tas/txae151

**Published:** 2024-10-23

**Authors:** Lydia K Olagunju, David P Casper, Uchenna Y Anele

**Affiliations:** Department of Animal Sciences, North Carolina Agricultural and Technical State University, Greensboro, NC 27411, USA; Department of Animal Sciences, North Carolina Agricultural and Technical State University, Greensboro, NC 27411, USA; Casper’s Calf Ranch, LLC, Freeport, IL 61032, USA; Department of Animal Sciences, North Carolina Agricultural and Technical State University, Greensboro, NC 27411, USA

**Keywords:** calves, growth performance, times feeding, milk replacer

## Abstract

Several calf studies demonstrated growth advantages when feeding greater protein and/or milk solids amounts, however, studies comparing 2 times per day vs. 3 times per day feeding are limited. The study objective was to evaluate feeding 2 times per day vs. 3 times per day thereby increasing milk solids intake. Forty 2- to 5-d old Holstein bull calves were blocked by body weight (**BW**) and randomly assigned to 1 of 2 treatments (*N* = 20/treatment) using a randomized complete block design. Treatments were 1) 2×: milk replacer (**MR**) fed 2 times per day; and 2) 3×: MR fed 3 times per day for increased solids intake. The MR was fed at 15% solids at 0630 and 1800 hours and the 3rd 3× feeding was at 1200 hours. Calves on 2× were fed MR at 0.567 kg/d for days 1 to 14, increased to 0.85 kg/d for days 15 to 35, and reduced to 1 time per day at 0.425 kg/d for days 36 to 42 to facilitate weaning at 42 d. Calves on 3× were fed MR 0.851 kg/d for days 1 to 14, increased to 1.275 kg/d for days 15 to 35, and reduced to 1 time per day at 0.425 kg/d for days 36 to 42 followed by weaning. Calf starter (**CS**; amounts and orts weighed daily) and water were offered for ad libitum intake. Initial BW was not a significant covariate and final BW (81.5 and 80.9 kg, for 2× and 3×, respectively) was similar. A treatment-by-week interaction (*P* < 0.01) indicated variable BW gains during the study with calves fed 3× demonstrating greater BW during weeks 4 (*P* < 0.10) and 5 (*P* < 0.05) compared with calves fed 2×. However, BW gains for calves fed 3× were reduced (*P* < 0.05) during the weaning period resulting in similar BW gains (36.8 and 36.4 kg) at the study end. Intake of CS (0.65 and 0.46 kg/d) was lower (*P* < 0.04) for calves fed 3× compared with calves fed 2×. Feed conversions (0.64 and 0.58 kg/kg) were greater (*P* < 0.02) for calves fed 2× compared with 3× fed calves. Calves fed 2× had less (*P* < 0.01) scours (fecal score = 0; 34.3 and 29.7 d) compared with 3× fed calves. Providing an additional MR feeding demonstrated minimal BW gains due to lower CS intake. Providing greater MR intake using a 3rd daily feeding reduces CS intake that can inhibit transition to dry feed when weaning calves.

## Teaser

Feeding neonatal calves milk replacer 3 times per day has become more common on dairy operations. This study demonstrated minimal growth improvements when fed milk replacer 3 times per day due to a slower transition to dry feeds during the weaning period.

## Introduction

Raising healthy and nutritionally efficient calves in a humane and cost-effective way is required to ensure the sustainability and efficiency of the global dairy industry (Fischer et al., 2019; van Niekerk et al., 2021). Bai et al. (2020) noted that successful calf-rearing programs are aimed to achieve optimal growth performance while achieving improved lactational performance upon calving. Historically, the amount of milk or milk replacer (**MR**) offered to calves has been restricted to maintain low rearing and labor costs (Kehoe et al., 2007). Several studies (Cowles et al., 2006; Guindon et al., 2015; Chapman et al., 2016) have focused on feeding recommendations for appropriate neonatal calf nutrition, although van Niekerk et al. (2021) noted major knowledge gaps in several critical developmental stages.

Calves will drink large amounts of milk if provided ( Jasper and Weary, 2002; Vieira et al., 2008; Borderas et al., 2009). The large amounts are reported to be close to their natural intakes (Mirzaei et al., 2018) which has supported recommendations for increasing protein and milk solids amounts to increase calf growth performance and health (Schingoethe et al., 1986; Kmicikewycz et al., 2013; Mirzaei et al., 2018; Jaeger et al., 2020b). Klopp et al. (2020) specifically highlighted increasing the milk or MR amount fed to dairy calves to ensure healthy animals with rapid and efficient prepubertal growth.

During the early days of a calf’s life, energy, and protein consumption influence growth and rumen development. Rapid rumen development can enhance the transition to dry feed. This developmental period is vital and critical to a neonatal calf’s growth performance and health (van Niekerk et al., 2021). The reported susceptibility to nutritional scours and other digestive problems can occur when calves are fed high milk amounts (Knauer et al., 2018; Fischer et al., 2019). Therefore, preweaning nutrition is important when neonatal calves are highly susceptible to health challenges (van Niekerk et al., 2021), even though most rearing strategies aim to achieve sufficient frame size and organ development without compromising future production (Heinrichs et al., 2017; Van Amburgh et al., 2019; Erickson et al., 2020; van Niekerk et al., 2021). Jaeger et al. (2020a) proposed optimal MR feeding rates with increasing CP amounts for enhancing lean tissue growth using a standard weaning program. Studies have focused on alternative milk-feeding methods to improve dairy calf growth performance and health without adverse effects on rumen development (Eckert et al., 2015; Omidi-Mirzaei et al., 2015; Mirzaei et al., 2017). van Niekerk et al. (2021) stated that determining the appropriate preweaning energy and protein balance supplied in both liquid and solid feeds indicated great potential for improving weaning growth and health. The frequency and amount at feeding time remain critical for transitioning from a milk-based diet to solid feed during rumen development.

Klopp et al. (2020) highlighted the increasing interest in the milk and/or MR amount fed to dairy calves to ensure healthy, but rapid and efficient prepubertal growth. Accelerated calf programs feeding relatively large milk volumes have become increasingly used by dairy producers (Welboren et al., 2019). However, these programs typically result in lower calf starter (**CS**) intakes during the transition period (weaning) to dry feed (Cowles et al., 2006; Guindon et al. 2015; Jaeger et al., 2020) and gradual weaning programs are not practical in most dairy operations unless using computerize feeding systems (Jaeger et al., 2020). Literature data on feeding frequency is lacking, but Schingoethe et al. (1986) recommended 2 or more liquid feedings for optimum calf growth and health. Grice et al. (2020) evaluated feeding frequency as 2 vs. 3 meals daily, but the MR CP concentrations were of concern.

The majority of dairy calves are fed 2 times per day (van Niekerk et al., 2021), which is in contrast to naturally occurring multiple feedings (Kmicikewycz et al., 2013). Therefore, feeding frequency, especially feeding times per day becomes increasingly important when greater volumes of milk are offered. Meanwhile, conflicting results were found to be reported on the growth performance of calves fed greater liquid feed volumes when compared with calves fed conventionally (Klopp et al., 2020). Hill et al. (2016) reported that feeding >0.66 kg MR (DM) could have a negative impact on digestibility, but feeding frequency may have future metabolic consequences by susceptibility to insulin resistance (Bach et al., 2013a, 2013b). Thus, increasing feeding frequency to reduce feeding amounts but increase total daily fed amounts may be beneficial for calves in first weeks of life to enhance growth and development while preventing/reducing health challenges, such as nutritional scours. Kmicikewycz et al. (2013) evaluated increased feeding frequency evaluating both conventional and a modified MR with the same MR quantity offered with a feeding frequency of 2 times per day vs. 4 times per day. This approach may not be practical for a dairy operation feeding calves, which supports the need for further research (Jensen et al., 2020).

Progressive dairy farm operations have transitioned to increase the feeding frequency of dairy heifers from 2 to 3 times per day when feeding of MR and/or whole milk to improve neonatal calf growth rates due to greater solids intake. While literature is more abundant on increasing the MR/milk amounts fed to enhance growth, there is limited literature on the impacts of increasing feeding frequency on calf growth performance. The hypothesis is that feeding calves greater MR amounts by increasing feeding frequency from 2 to 3 times per day will improve growth performance. The study objective is to evaluate feeding MR 2 vs. 3 times/d which will not only increase feeding frequency, but amounts fed.

## Materials and Methods

The following experiment reported here was conducted using the same or similar facilities, experimental design, procedures, treatment protocols, and sample collections as previously reported experiments by this Contract Research facility (Bai et al., 2020; Liu et al., 2020; Casper et al., 2021). The specific materials and methods are briefly reported below.

### Calf Management and Feeding

The experiment was conducted at Casper’s Calf Ranch (Freeport, IL) starting on April 6, 2021, and ending on June 1, 2021, for a 56-d (8 wk) experimental period. The calves were managed and fed according to the guidelines published in the 4th edition “Guide for the Care and Use of Agricultural Animals in Research and Teaching” published by ADSA-ASAS-PSA (2020). In addition, the experimental protocol and all treatment protocols were reviewed by attending licensed veterinarians (Lena Veterinary Clinic, Lena, IL). Forty Holstein bull calves being 2- to 5-d were sourced through a commercial calf buyer (Knueppel Livestock, Shawno, WI) and delivered on Monday night (April 5, 2021). Calves were purchased through several livestock auction barns and represented commingled calves sourced from numerous dairy farms. Calves were assumed to have been fed colostrum at the originating dairy operations, but the colostrum program on each dairy operation is unknown. One 10-mL blood sample was collected on April 7 during the afternoon via jugular venipuncture (Becton Dickinson Vacutainer Systems, Rutherford, NJ) by licensed veterinarians (Lena Veterinary Clinic, Lena, IL), placed on ice, centrifuged at 2,000 × *g* for 10 min at room temperature for measurement of total serum protein (**TSP**) concentrations using a clinical refractometer (Model 300005, SPER Scientific, Scottsdale, AZ). Calves were vaccinated upon arrival with Inforce 3 (Zoetis Inc. Florham Park, NJ) and placed in a chopped wheat straw bedded calf hutch (Calf-Tel Deluxe II, Hampel, Germantown, WI) measuring 220 *×* 122 *×* 38 cm that was placed on a grass pasture in an open naturally well-ventilated area. Hutches were spaced 0.6 m apart in rows of 10 hutches. Each hutch had a 183 cm × 122 cm × 107 cm wire panel attached to the front with 2 bucket holders (0.65 m high) for 8 L plastic buckets. One bucket contained ad libitum fresh water and the other bucket contained a pelleted CS. The water bucket was emptied at each feeding and filled with the appropriate experimental MR at the designated feeding time. Upon completion of milk consumption, the bucket was rinsed and filled with fresh water.

Calves were fed the 22/20 (CP:Fat) MR with 41.7 mg/kg “As is” decoquinate (Deccox, Zoetis Inc., Parsippany, NJ) and 16 mg/kg diflubenzuron (ClariFly, Central Life Sciences, Schaumburg, IL), the next morning after arrival during the night. The 22/20 MR has the AA composition of 24/20 using synthetic AA that are commercially available. The MR ingredient composition is given in Table 1. After the morning feeding, calves were weighed, and frame measurements were collected. Calves were then blocked by BW and randomly assigned to 1 of 2 MR treatments using a randomized complete block design (**RCBD**). Treatments were 1) Control: Calves fed MR 2 times per day (2×) at 6:30 am and 6 pm, and 2) Calves fed MR 3 times per day (3×) with the additional feeding at noon.

**Table 1.** Ingredient composition of 22% crude protein (22; CP As Fed) MR containing the essential amino acid composition equal to 24% CP^1^

	Milk Replacer
Ingredient	22
	--- (% of Mix) ---
Whey, whey protein concentrate	49.44
Fat base	45.28
Dicalcium phosphate	0.85
l-Lysine HCL	0.99
Decoquinate 0.5% (Deccox)^2^	1.00
Vitamin premix	0.94
dl-Methionine	0.46
Organic mineral mix	0.38
Diflubenzuron (Clarify 8%)^3^	0.02
Flavor	0.06
Threonine	0.27
l-Valine	0.12
Vitamin E	0.09
l-Leucine	0.12

^1^Tag guarantee of 22% crude protein, 20% fat, not less than 55,000 international units (**IU**) of Vitamin A, 16,500 IU of vitamin D, and 220 IU of vitamin E per kg on an “As Fed” basis.

^2^Zoetis Inc., Florham Park, NJ.

^3^Central Life Sciences, Schaumburg, IL.

Each MR was accurately weighed (Model ACE110, Smart Weigh Inc., Hurricane, WV) and mixed with the appropriate amount of 46 °C hot water using an Urban Milk Shuttle (Urban GmbH & Co., Hamburg, Germany). The shuttle with its computerized mixing, heating, and delivery systems was calibrated to ensure an accurate delivery of a homogeneously mixed MR being fed at a temperature of 37.5 °C or greater at the correct volume. The MR was fed at the rate of 0.57 kg/calf daily for the first 14 d, then increased to 0.85 kg/calf daily divided into 2 equal feedings (i.e., morning and evening) for calves on 2×. Milk replacer was fed at the rate of 0.855 kg/calf daily for the first 14 d, then increased to 1.275 kg/calf daily divided into 3 equal feedings (i.e., morning, noon, and evening) for calves fed 3×. The shuttle is rinsed after feeding each treatment. For 5 d during week 5 (days 30 to 35) amprolium (Corrid, Merial, LLC., Duluth, GA) was added at the rate of 10 mg/kg BW to the MR for coccidiosis control. Starting on days 36 to 42, the MR feeding rate was decreased to 0.425 kg/calf daily fed 1 time per day at the morning feeding to facilitate weaning for the 40 calves. All MR were fed at 15% solids and if any calf did not consume its milk, the refused volume was recorded. Starting on day 1, a 2.4-mm mini-pellet pelleted 22% CP CS (as is basis; Table 2), and water was offered ad libitum throughout the study. The MR was manufactured and supplied by Milk Specialties (Eden Prairie, MN) and CS was formulated by Casper Ranch, manufactured, and supplied by a commercial feed company (ADM, Quincy, IL). All MR and CS were manufactured in sufficient quantities at one time to complete the study using the same lots of ingredients (Tables 1 and 2).

**Table 2.** Ingredient composition of pelleted CS

Ingredient	Calf starter (% of mix)
Wheat midds	34.95
Soybean meal, 47.5%	25.80
Corn, fine ground	22.50
Molasses mixer	5.50
Corn distiller’s grains with solubles	5.00
Calcium carbonate	2.25
Wheat starch	1.25
Salt	1.10
Bentonite	0.50
Soy oil	0.45
CitriStim^1^	0.25
Selenium yeast 600	0.11
Decoquinate, 6%^2^	0.08
Choline chloride 70	0.07
Trace mineral premix^3^	0.06
Trace mineral and vitamin premix^4^	0.06
Flavor, Covotek 570^5^	0.03
Niacinamide	0.02
Vitamin A/D premix^6^	0.02
Thiamin mononitrate 100%	0.01
Vitamin B_12_ 0.1%	0.0025

^1^CitriStim is *Pichia guilliermondil* yeast, ADM Animal Nutrition, Decatur, IL.

^2^Central Life Sciences, Schaumberg, IL.

^3^Trace mineral premix contains cobalt 2,800 mg/kg, copper 4,000 mg/kg, iodine 4,950 mg/kg, manganese 16,000 mg/kg, and zinc 16,000 mg/kg.

^4^Trace mineral and vitamin premix contains copper 82,910 mg/kg, manganese 45,460 mg/kg, 250,000 mg/kg, vitamin A 1,984,140 IU/kg, vitamin D 354,941 IU/kg, vitamin E 154,322 IU/kg, choline 258,200 mg/kg, biotin 502 mg/kg, folic acid 5,490 mg/kg, pantothenic acid 60,100 mg/kg, 10,000 mg/kg riboflavin and vitamin B_12_ 95,700 mg/kg.

^5^Flavor, ADM Animal Nutrition, Decatur, IL.

^6^Contains 11,937,909 vitamin A and 4,268,106 IU/kg vitamin D, respectively.

### Weather Data

Weather data were downloaded from a local personal weather station site for Freeport, IL located at 42.301°N, 89.665°W at an elevation of 256.9 m being approximately 5 km from the research location. The temperature-humidity index (**THI**) was calculated according to the equation of (Vitali et al., 2009) based on the daily weather station data for minimum, average, and maximum THI values. A THI value of <72 was considered no heat stress, 72 to 78 mild, 79 to 88 moderate, and >89 as severe heat stress. A THI value of >78 would be considered to compromise the calf’s welfare. These HS ranges are based on limited literature data (Roland et al., 2016; Kovács et al., 2020) and the thermoneutral zone (TNZ) of 13 to 25 °C is based on TNZ for a 1 mo old calf according to Wang et al. (2020).

### Feed Intake and Analysis

Starting on day −1, the CS weights offered and refused were recorded daily using a digital scale (Model ACE110, Smart Weigh Inc.). In case of days when feed was wet, (i.e., rain), that day’s data were eliminated and the remaining days during that week were compiled into weekly means of CS intake. There were 10 d when rain prevented feed intake measurement and 3 d was the minimum used for calculating a weekly mean (4 d lost for week 1, 3 d lost for week 4, 2 d lost for week 6, and 1 d lost for week 7). Samples of each experimental MR and CS were collected weekly and stored frozen at −20 °C until composited at the end of the study. Samples collected during weeks 1, 2, and 3 or weeks 4, 5, and 6 were composited into 2 samples for the MR, while CS samples collected during weeks 1, 2, 3, and 4 or weeks 5, 6, 7, and 8 were composited into 2 CS samples. Samples of MR and CS were submitted to Dairyland Laboratories (Arcadia, WI) for nutrient analyses. Samples of MR were analyzed using the following AOAC International (2019) methods for DM (930.15), CP (990.03), acid hydrolysis fat (954.02), NDF (2002.04), ADF (973.18), ash (942.05), Ca (985.01), P (985.01), Mg (985.01), K (985.01), S (923.01), Na (985.01), Cu (985.01), Fe (985.01), Mn (985.01), and Zn (985.01). Calf starter samples were analyzed for DM (930.15), CP (990.03), soluble protein (Krishnamoorthy et al., 1982), NDF (2,002.04), ADF (973.18), lignin (973.18), NDF-insoluble protein (2,002.04 without sulfite and 976.06), ADF-insoluble protein (973.18 and 976.06), water-soluble sugar (DuBois et al., 1956), starch (Hall, 2009), fat (2,003.05), ash (942.05), Ca (953.01), P (953.01), Mg (953.01), K (953.01), S (953.01), Na (953.01), Al (953.01), B (953.01), Cu (953.01), Fe (953.01), Mn (953.01), and Zn (953.01). Chloride for both MR and CS samples was extracted with 0.5% nitric acid and analyzed by potentiometric titration with silver nitrate (Metrohm 848 Titrino Plus, Metrohm, Riverview, FL). Nonfiber carbohydrates were calculated using the equation of: NFC = [100 – ((NDF – NDF-insoluble protein) + CP + fat + ash)], while ME (Mcal/kg) was calculated for MR and CS using the equations of Quigley (2007) and NRC (2001).

Milk replacer solubility was evaluated by mixing a typical MR feeding of 0.425 kg with 2.85 L of 43.3°C water and sitting for 5 to 10 min (approximate consumption time by calf) and then filtered through 4 layers of cheesecloth. The cheesecloth was dried and weighed before and after filtering the individual MR samples to determine the amount of residue remaining on the cheesecloth. The 2 experimental composite MR samples were replicated 7 times.

### Body and Health Measurements

Body weight was measured weekly using a Wrangler Jr. digital scale (Digi-Star, LLC, Fort Atkinson, WI) placed on a 1.2 m × 2.4 m sheet of 1.9 cm thick sheet of plywood towed by a John Deere 825 Gator (John Deere, Moline, IL). Body weights were taken after the morning feeding starting at approximately 0900 hours each week. Hip height and WH were measured using a Ketchum Teletape having a level mounted on top (Ketchum Manufacturing Inc., Brockville, ON, Canada); BL and HG were measured using a Nasco dairy calf weigh tape (Nasco, Fort Atkinson, WI). Body measurements were taken at the same time as BW, but only 0 and 8 wk (i.e., weeks 0 and 8).

Calf health along with fecal, nasal, and ear/eye scores were monitored daily during the experiments. Health scores were visually assessed before the evening feeding according to the University of Wisconsin calf health scoring chart (McGuirk, 2013) and were based on a scale of 0 to 3. Fecal scores were established as 0) normal, 1) semi-formed and/or pasty, 2) loose but stays on top of bedding, and 3) watery and/or sifts through bedding. Nasal scores were 0) normal serous discharge, 1) small amount of unilateral cloudy discharge, 2) bilateral and/or cloudy or excessive mucus discharge, and 3) copious bilateral mucopurulent discharge. Eye/ear scores were 0) normal, 1) small amount of ocular discharge with ear flick or head shake, 2) moderate amount of bilateral ocular discharge and/or slight unilateral droop, and 3) heavy ocular discharge and/or head tilt or bilateral droop. In cases of illness, body temperature was measured using a rectal thermometer (Zoe + Ruth, Portland, OR) and appropriate medical treatments as prescribed by a licensed veterinarian (Dr. Brandon Scharping, Lena, IL) were administered if needed. All health incidents and treatments were recorded during the study.

### Statistical Analysis

Several power analyses have been conducted across our studies using prior means and variation, as well as a current poststudy evaluation. The power analysis indicated that a minimum of 13 to 15 calves per treatment being required to detect significant differences at the *P* < 0.05 level with 80% power for the key study growth parameters (i.e., BW gain, average daily gains [ADG], dry matter intake [DMI], and feed conversions). Therefore, a safety factor of 5 additional calves/treatment has been included in the studies to account for variation among calves from different dairy operations, different sale barns, inability to source replacements if a calf is lost, and potential outliers resulted in sourcing a minimum of 20 calves per treatment. Thus, 1 or 2 calf deaths or an outlier will not influence the experiment’s ability to detect treatment differences. The current death loss is <2% among several experiments which have been conducted at this research facility.

All data were checked for normality and outliers using the UNIVARIATE procedure of SAS (version 9.4, SAS Institute, Cary, NC) before any statistical analyses were conducted. The box and whisker plots and the Shapiro Wilk Test were used to verify that the remaining data were normally distributed (*P* > 0.15). One calf died (discussed later) while 39 calves finished the 56 d experiment with no identified outliers. All data were then subjected to least squares analysis of variance (ANOVA) for a RCBD (Steel & Torrie, 1980) having 2 treatments via the MIXED procedure of SAS with study week as a repeated measure ANOVA. The statistical model used was:


$$\begin{array}{l} Y_{ijk}=\mu +Rep_{i}+Trt_{j}+Wk_{k}+\left(Trt_{j}\times \;Wk_{k}\right)+Cov+e_{ijk} \end{array}$$

Where Y_*ijk*_ = dependent variable, µ= overall mean, Rep_*i*_* *= replication or block, Trt_*j*_ = treatment, Wk_*k*_ = week of study, Trt_*j*_ × Wk_*k*_ = treatment-by-week interaction, Cov = Covariate (initial measurement when appropriate), and e_*ijk*_ as the residual random error. Treatment, week, and treatment-by-week interaction were considered fixed effects, while replication was considered a random effect. Calf is considered random although not explicitly coded in the model. Study week was considered a repeated measurement in time having an autoregressive covariance structure. Least squares means were separated by PDIFF statement when the *F*-test for treatment was significant (*P* < 0.05). All other data were summarized utilizing the model described above but excluded week. Initial BW and frame measurements were tested as a covariate for their respective parameter but did not improve statistical significance (*P* > 0.15) and therefore were excluded from the model. Significance was declared at *P* < 0.05 and trends at 0.05 < *P* ≤ 0.10. Daily feed intake and orts measurements were compiled as weekly averages and DM intakes were calculated. Each daily fecal, nasal, and eye/ear scores were summarized by tallying by week the number of days having a specific score, (i.e., number of days of score 0), and analyzed as weekly averages and the second way was to total the number of days of a specific score for the entire 6-wk MR phase of the study.

## Results and Discussion

### Milk Replacer Mixability

The residue amount remaining after mixing a typical feeding (approximately 0.43 kg) with 110 °C water was 1.14% ± 0.48 which was the same MR for calves fed MR at 2 and 3 times per day. Mixability is always a concern with MR because residues left in the container after feeding are a concern both to the calf for not being able to consume all of the allotted nutrients and the person feeding the calves who notices the residue remaining. The MR mixed easily and stayed in suspension to ensure accurate and precise feeding.

### Milk Replacer and Calf Starter Nutrient Composition

The MR was formulated according to the recommendations of Bai et al. (2020) to have the amino acid concentrations of a 24% CP MR, but CP reduced to 22% CP (as fed basis, > 23% CP DM basis) recommendation by using synthetic amino acids (Table 1). The MR nutrient composition indicated that the nutrient composition met or slightly exceeded formulated specifications for CP and minerals (Table 3).

**Table 3.** Nutrient composition of MR and pelleted CS^1^

	Milk		Calf	
Nutrient	Replacer	SD	Starter	SEM
*N* ^2^	2	—	2	—
DM^3^, %	97.5	0.17	88.2	0.49
CP^4^, %	24.0	0.26	24.3	0.35
SP^5^, % of CP	—	—	32.7	4.40
ADF^6^, %	0.08	0.03	7.92	0.28
NDF^7^, %	0.28	0.08	18.5	0.57
ADF-ICP^8^, %	—	—	0.59	0.05
Soluble fiber, %	—	—	9.00	0.23
Lignin, %	—	—	1.57	0.27
NFC^9^, %			46.3	0.93
Starch, %	—	—	25.1	0.58
Fat, %	20.5	0.09	3.95	0.06
ME^10^, Mcal/kg	20.7	0.08	2.95	0.01
NE_m_^11^, Mcal/kg	—	—	1.99	0.01
NE_g_^12^, Mcal/kg	—	—	1.34	0.01
Ash, %	7.55	0.07	8.99	0.18
Ca, %	1.13	0.01	1.38	0.10
P, %	0.85	0.01	0.79	0.02
Mg, %	0.12	<0.01	0.29	0.01
K, %	1.59	0.01	1.53	0.04
S, %	0.45	0.03	0.27	0.01
Na, %	0.71	<0.01	0.46	0.05
Cl, %	1.11	0.04	0.80	0.06
Boron, ppm	3.00	<0.01	9.0	<0.01
Al, ppm	34.5	6.36	573.5	16.3
Fe, ppm	141.0	19.8	357.5	9.19
Mn, ppm	53.0	2.83	212.0	12.7
Zn, ppm	137.0	25.5	233.0	18.4
Cu, ppm	12.0	<0.01	75.0	11.3

^1^Analyses conducted by Dairyland Laboratory, Arcadia WI.

^2^ *N* = number of samples.

^3^Dry matter.

^4^Crude protein.

^5^Soluble protein.

^6^Acid detergent fiber.

^7^Neutral detergent fiber.

^8^Acid detergent fiber insoluble crude protein.

^9^Non-fiber carbohydrate.

^10^Metabolizable energy.

^11^Net energy maintenance.

^12^Net energy gain.

The mini-pelleted CS nutrient concentrations were slightly below formulation specifications of a 22% CP (As fed; 24.3% CP DM) while the remaining nutrients met or exceeded formulated specifications. These nutrient concentrations would meet or exceed the nutrient requirement guidelines for growing neonatal Holstein dairy calves (NRC, 2001; NASEM, 2021).

### Weather Data

The maximum, average, and minimum daily temperatures and calculated THI fluctuated during the period (Table 4) with the average maximum value of 65.8 for THI. A THI < 78 has been reported not to compromise calf welfare according to (Kovács et al., 2020), since calves were not exposed to heat stress (THI > 78). Maximum THI for week 7 was 78.7, which was above the lowest upper critical THI associated with heart rate. But the minimum for the week was 57.9, which had an impact on the average THI for the week with a value of 68.7. Therefore, feed intake and the immune system are not expected to be negatively impacted (Wang et al., 2020). During the study, the maximum weekly temperatures were within the thermoneutral zone (TNZ) range (i.e., 13 to 25 °C) described by Wang et al. (2020) for calves within 1 mo of age. The maximum temperature for week 7 was 26.7 (>25 °C) with subsequent increase in rainfall. The minimum for the week was 12.6 and the average for the week was 21.5, which were not expected to negatively impact calf growth. Maximum, average, and minimum wind speed were normal (>5 mph), which were favorable for the calves’ welfare. The amount of precipitation received during the study was 7.7 cm, indicating basically that the late 2021 Spring was wet.

**Table 4.** Weather data for the 8-wk experimental period

	Temperature, °C			Humidity, %			Wind speed, km/h			THI, °C^1^			Rain, cm	
Week	Max	Mean	Min	Max	Mean	Min	Max	Mean	Min	Max	Mean	Min	Mean	Total
1	18.0	14.1	10.5	84.4	72.1	54.3	30.6	15.8	3.7	63.5	57.2	52.6	0.25	1.73
2	13.7	8.5	3.2	73.9	52.0	34.6	26.0	14.5	3.7	57.0	50.1	45.0	0.00	0.00
3	15.1	9.6	3.7	64.4	45.7	30.3	30.3	14.7	2.8	58.8	51.9	46.1	0.00	0.00
4	25.4	18.3	12.1	71.6	51.8	33.6	33.6	17.7	6.7	74.5	63.0	55.6	0.01	0.05
5	15.8	10.6	5.4	79.9	56.8	35.7	25.3	13.5	0.0	60.2	52.8	47.6	0.36	2.49
6	19.6	13.9	7.4	80.3	54.9	32.6	19.1	8.1	0.0	66.2	57.4	50.6	0.05	0.36
7	26.7	21.5	12.6	88.4	73.0	45.3	26.4	13.5	2.5	78.7	68.7	57.9	0.21	1.50
8	20.5	15.6	10.3	80.6	60.4	40.1	28.3	16.1	6.2	67.6	59.5	53.1	0.24	1.65
Mean	19.4	14.0	8.1	77.9	58.3	38.3	27.4	14.3	3.2	65.8	57.6	51.1	0.14	7.77
SD	6.39	5.34	6.62	9.30	13.06	14.7	8.35	5.65	4.66	10.02	7.39	6.89	0.35	0.35

^1^Temperatue-humidity index.

### Growth Performance

**Figure 1.**
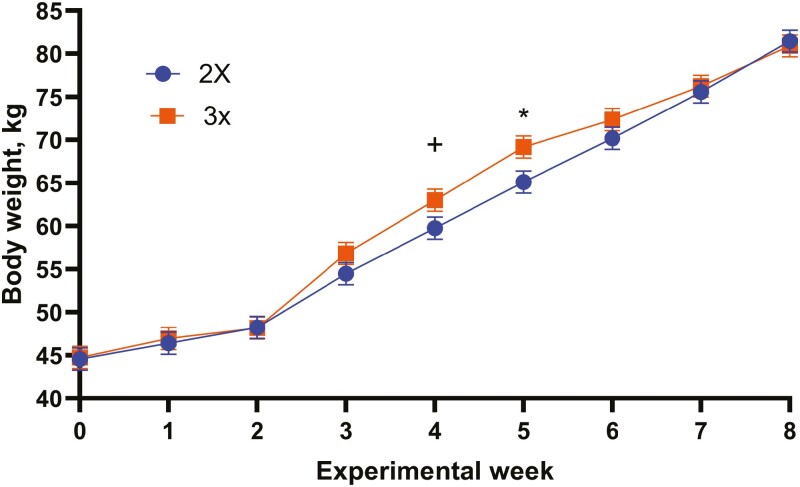
Body weight (kg) for calves fed MR 2 (2×) or 3 (3×) times per day. Treatment by week interaction, *P* < 0.01. SE = 1.28. ^+^*P* < 0.10. ^*^P < 0.05.

The study was initiated with 20 calves/treatment, but 1 calf died due to respiratory disease (Table 5), which was assumed to be unrelated to treatment. There was a treatment-by-week interaction (*P* < 0.01) indicating that calves fed 3 times per day was similar in growth to calves fed 2 times per day during the first 2 wk of the study, but then demonstrated increased BW gains during weeks 4 (*P* < 0.10) and 5 (*P* < 0.05), but then decreased back to similar BW growth during weeks 6, 7, and 8 (Figure 1). Providing an additional MR feeding demonstrated minimal BW gains (Table 5) which are in contrast to data reported by Korst et al. (2017) that restricting the amount of liquid feed results in lower growth rates. Although there was an increase (*P* < 0.05) in BW when calves were fed 3× at week 5 compared with calves fed 2×, calves fed 2× were able to catch up with the preweaning differences in BW postweaning. The early weaning protocol resulted in a reduction in growth rate for the 3× calves due to lower CS intake. These data are not in an agreement with our hypothesis. Even though increased consumption of milk/MR based on previous studies (Cowles et al., 2006; Guindon et al., 2015; Chapman et al., 2016) and recommendations was hypothesized to improve the 3× growth rate as discussed earlier. Although, Klopp et al. (2020) noted that calf growth is significantly affected by the preweaning diet, as well as the transitional steps used to wean calves.

**Table 5.** Total serum protein, BW and ADG for calves fed MR 2 (2×) or 3 times (3×) per day

	Treatment (Trt)			*P* < ^1^	
Measurement	2×	3×	SEM	Trt	Trt*Week
Calves, #	19	20	—	—	—
TSP, g/dL	5.2	5.2	0.14	0.85	—
BW, kg					
Week 0, initial	44.6	44.8	1.28	0.92	0.01
Week 1	46.4	46.9	1.28		
Week 2	48.2	48.2	1.28		
Week 3	54.5	56.8	1.28		
Week 4	59.8	63.0^+^	1.28		
Week 5	65.1	69.2^*^	1.28		
Week 6	70.2	72.3	1.28		
Week 7	75.6	76.2	1.28		
Week 8, final	81.5	80.9	1.28		
Study average	60.6	62.0	1.17	0.34	—
Study BW gain, kg	36.8	36.4	1.89	0.85	—
ADG, g/d					
Week 1	263.9	314.7	72.2	0.69	0.01
Week 2	259.4	179.0	72.2		
Week 3	892.5	1241.8^*^	72.2		
Week 4	758.7	884.7	72.2		
Week 5	764.7	884.7	72.2		
Week 6	724.1	454.7^*^	72.2		
Week 7	772.2	557.5^*^	72.2		
Week 8	979.7	784.9^*^	72.2		
Study average	676.9	662.6	29.8	0.69	—
Study 0 to 56 d	668.9	661.0	34.3	0.85	—

^1^Probability of *F*-test for treatment and treatment-by-week interaction.

^+^ *P* < 0.10.

^*^ *P* < 0.05.

**Figure 2.**
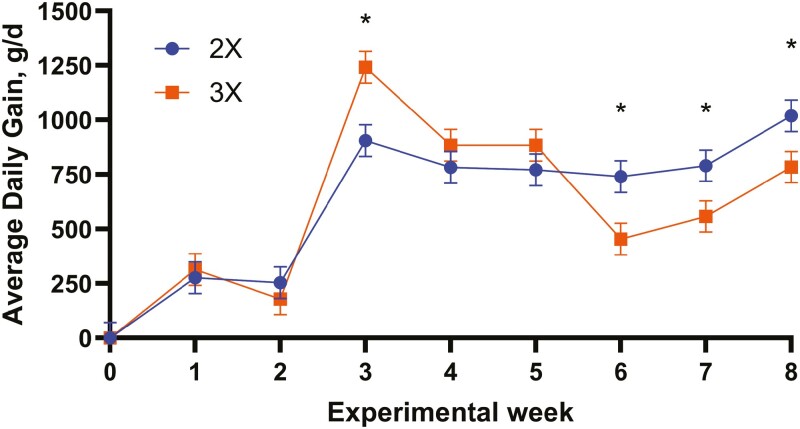
Average daily gain (g/d) when fed MR 2 (2×) or 3 (3×) times per day. Treatment by week interaction, *P* < 0.01. SE = 72.2. ^+^*P* < 0.10. ^*^P < 0.05.

The ADG for calves fed 3× was greater (*P* < 0.05) at week 3 (Table 5; Figure 2), which could be associated with the increased level of MR nutrient intake as reported by (Cowles et al., 2006; Knauer et al., 2018), which is agreement with Jensen et al. (2020). Jensen et al. (2020) explained that before the age of 3 to 4 wk, the calf has limited capacity to digest solid feeds, consequently growth performance relies primarily on milk nutrients to meet the nutrient requirements for maintenance and growth, which increases preweaning ADG when offered high amounts of milk/MR.

However, the current study demonstrated no statistical improvement in ADG for calves fed 3× compared to calves fed 2× by week 4, even though it was numerically higher but remained constant during week 5 while calves fed 2× were numerically improving. Eventually, calves fed 3× dropped (*P* < 0.05) in ADG during week 6 compared to calves fed 2×. Calves fed 2× demonstrated increases (*P* < 0.05) in ADG until the end of the study compared to calves fed 3×. The present study findings are not in agreement with performance previously report by (Khan et al., 2007) on higher pre- and postweaning ADG for calves who ingested an average of 7.7 L/d of milk. Similarly, these data are not in support of Hill et al. (2016) who reported that calves fed > 0.7 kg of dry matter from MR typically have greater growth performance preweaning because calves’ growth performance was expected to improve with higher amounts of MR. But the study confirms an insight provided by Welboren et al. (2019) on calf growth potentially being limited during weaning by metabolizable energy (**ME**) intake rather than CP intake.

### Dry Matter Intake and Feed Efficiency

**Figure 3.**
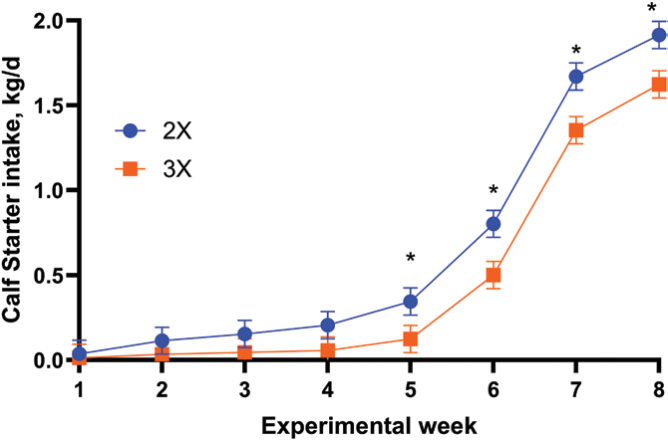
Calf starter intake (kg/d) when fed MR 2 (2×) or 3 (3×) times per day. Treatment by week interaction, *P* < 0.10. SE = 0.08. ^*^P < 0.05.

**Figure 4.**
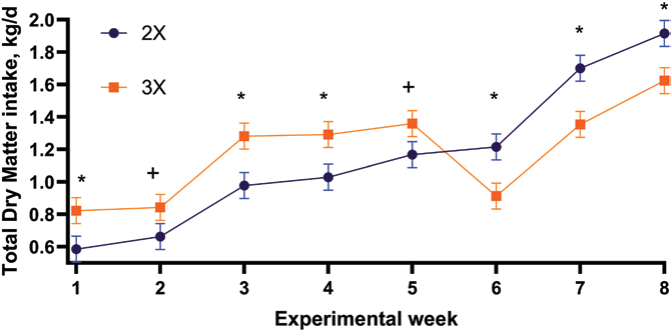
Total DMI (kg/d) when fed MR 2 (2×) or 3 (3×) times per day. Treatment by week interaction, *P* < 0.01. SE = 0.08. ^+^*P* < 0.10. ^*^P < 0.05.

Per experimental design, MR DMI was greater (*P* < 0.01) for calves fed 3× compared with calves fed 2× throughout the preweaning period (Table 6). Study average CS DMI was approximately 63% lower (*P* < 0.04) for calves fed 3× compared with calves fed 2× (Table 6). There was a trend (*P* < 0.10) for a treatment-by-week interaction demonstrating that calves fed 3× were consuming approximately 50% of the CS being consumed by calves fed 2× (Figure 3). There was a treatment-by-week interaction (*P* < 0.01) for total (MR + CS) DMI (Table 6). The calves fed 3× were consuming greater total DMI during the early study weeks, but DMI decreased during the weaning and postweaning phases (Figure 4). The inability of calves fed 3× to transition to dry feed resulted in declining BW and ADG improvements during the later study weeks. These data demonstrate that feeding more MR DM as a 3rd feeding greatly reduces CS DMI, which can lead to a delay (inhibition) in transitioning from liquid to dry feed. These data agree with Jensen et al. (2020) in that lower milk allowance stimulated solids intake although this contrasts with Jensen et al. (2020) that larger milk meals stimulated appetite for solid feeds. Jaeger et al. (2020a) and Chapman et al. (2016) reported that MR feeding rate reduced CS intake which can result in a post-weaning growth slump.

**Table 6.** Dry matter intake (DMI) and feed conversions for calves fed MR 2 (2×) or 3 times (3×) per day

	Treatment (Trt)			*P* < ^1^	
Measurement	2×	3×	SEM	Trt	Trt*Week
Calves, #	19	20	—	—	—
MR DMI, kg/d					
Weeks 1 and 2	0.55	0.82^**^	< 0.01	0.01	0.01
Weeks 3, 4, and 5	0.82	1.25^**^	< 0.01		
Week 6	0.41	0.41^**^	< 0.01		
Total MR, kg	27.8	40.6^**^	< 0.01	0.01	—
CS DMI, kg/d					
Week 1	0.04	0.01	0.08	0.04	0.10
Week 2	0.11	0.03	0.08		
Week 3	0.15	0.05	0.08		
Week 4	0.20	0.06	0.08		
Week 5	0.34	0.12^*^	0.08		
Week 6	0.79	0.50^**^	0.08		
Week 7	1.68	1.34^**^	0.08		
Week 8, final	1.89	1.65^**^	0.08		
Study average	0.65	0.46^*^	0.07	0.04	—
Days to eating 0.9 kg for 3 d	38.9	42.9	1.18	0.01	—
Total DMI, kg/d					
Week 1	0.59	0.83^*^	0.08	0.71	0.01
Week 2	0.67	0.85^+^	0.08		
Week 3	0.98	1.29^*^	0.08		
Week 4	1.03	1.30^*^	0.08		
Week 5	1.17	1.36^*^	0.08		
Week 6	1.21	0.91^*^	0.08		
Week 7	1.67	1.34^*^	0.08		
Week 8	1.89	1.61^*^	0.08		
Study average	1.15	1.19	0.07		
Total DMI, 0 to 56 d	65.5	66.4	3.40	0.75	—
Gain/DMI, kg/kg					
Week 1	0.499	0.432	0.06	0.02	0.81
Week 2	0.428	0.265^*^	0.06		
Week 3	0.964	1.018	0.06		
Week 4	0.784	0.735	0.06		
Week 5	0.709	0.700	0.06		
Week 6	0.605	0.510	0.06		
Week 7	0.517	0.458	0.06		
Week 8	0.584	0.539	0.06		
Study average	0.636	0.581^*^	0.03	0.02	—
Study 0 to 56 d	0.638	0.591	0.03	0.03	—

^1^Probability of *F*-test for treatment and treatment-by-week interaction.

^+^ *P* < 0.10.

^*^ *P* < 0.05.

^**^ *P* < 0.01.

The reductions in CS and total DMI resulted in feed conversions being greater (*P* < 0.03) for calves fed 2× compared with calves fed 3×. These data confirm results by Welboren et al. (2019) that greater milk allowance delays CS intake, thus maintaining the same weaning objectives becomes challenging. The feed efficiency was significantly greater (*P* < 0.05) for calves fed 2× compared with calves fed 3× starting from week 2. This finding contrasts with Jensen’s et al. (2020) explanation that calves are unable to compensate for low milk intake during the first weeks of life because young calves cannot digest much concentrate, which creates a further concern that limited milk feeding may cause undernutrition. Likewise, the current study result is not in agreement with Klopp et al. (2019) that reported greater preweaning feed efficiency for calves fed higher levels of MR compared to moderate levels of MR. The improved feed efficiency for calves fed 2× might agree with the statement (Jensen et al., 2020) that feeding fewer MR portions results in better digestibility, including complete casein hydrolysis and curd formation. But it does not correspond with the concept (Kesser et al., 2017) regarding mechanisms underlying better future productive performance for calves reared on high levels of MR, which highlighted greater feed digestibility and better nutrient utilization from improved gastrointestinal function and liver metabolism.

### Frame Measurements

Feeding calves MR at 2× or 3× resulted in similar body frame measurements, except for withers height gain being greater (*P* < 0.04) for calves fed 2× compared with calves fed 3× (Table 7). Why withers height gain would be greater for calves fed 2× is not known, but could be related to the greater CS DMI or the reduction in total DMI for calves fed 3×. Several researchers (Cowles et al., 2006; Guindon et al., 2015; Chapman et al., 2016) reported greater frame measurements with greater MR feeding rates. This study demonstrates that feeding calves MR 3× feeding did not improve structural growth. Welboren et al. (2019) suggested that when feeding greater CP compared to ME intake indicates that energy supply may be more important compared to protein for the weaning and early post-weaning periods. Also, an abrupt weaning program could cause growth depression in calves around weaning due to severe loss and longer lag in fulfilling the gap between ME loss from milk to ME supply from solid intake (Khan et al., 2011, 2016; Welboren et al., 2021).

**Table 7.** Body frame measurements for calves fed MR 2 (2×) or 3 times (3×) per day

	Treatment (Trt)			*P* < ^1^
Measurement	2×	3×	SEM	Trt
Calves, #	19	20	—	
Hip height				
Initial, cm	81.7	81.7	0.82	0.99
Final, cm	92.8	92.4	0.92	0.76
Gain, cm	11.0	10.7	0.75	0.77
Hip width				
Initial, cm	20.9	20.9	0.22	0.95
Final, cm	25.8	25.8	0.31	0.98
Gain, cm	4.9	4.9	0.25	0.88
Withers height				
Initial, cm	77.1	77.9	0.74	0.41
Final, cm	88.4	87.5	0.78	0.37
Gain, cm	11.2	9.6	0.53	0.04
Heart girth				
Initial, cm	80.5	80.5	0.62	0.99
Final, cm	98.9	99.2	1.00	0.83
Gain, cm	18.4	18.6	0.93	0.82
Body length				
Initial, cm	55.1	55.1	0.76	0.96
Final, cm	64.8	65.3	0.77	0.30
Gain, cm	9.5	10.3	0.63	0.38

^1^Probability of *F*-test for treatment and treatment-by-week interaction.

### Health Performance

Calves fed 3× had greater (*P* < 0.01) scour scores compared with calves fed 2× whether expressed as total days or days per week (Table 8). Current data demonstrate that increasing the times/amount fed per day increased the number of calves having scours. Liu et al. (2020) reported that calves fed MR containing greater concentrations of synthetic EAA demonstrated more scours than calves fed lower concentrations of synthetic EAA. The use of synthetic EAA reduces inclusion rate of all milk protein ingredients allowing for increased NFC concentrations. The increased NFC concentration source would be from a greater lactose concentration. Davis and Drackley (1998) reported research that high lactose intakes can lead to an increased scours incidence. Whole milk on a DM basis would contain less than 40% to 45% lactose (NRC, 2001; Berends et al., 2020) thus increasing lactose concentrations could influence scour incidences. In agreement with reduced lactose concentrations influencing scours, Berends et al. (2020) reported that exchanging fat for lactose in MR reduced the number of health events.

**Table 8.** Fecal, nasal, and eye and ear scores for calves fed MR 2 (2×) or 3 times (3×) per day

	Treatment (Trt)			*P* < ^1^	
Measurement	2×	3×	SEM	Trt	Trt*Week
Calves, #	19	20	—	—	—
Total days of fecal scores					
Score = 0	34.3	29.7	0.99	0.01	—
Score = 1	4.6	6.7	0.61	0.01	—
Score = 2	1.9	4.2	0.50	0.01	—
Score = 3	1.2	1.5	0.40	0.55	—
Fecal scores, d/wk					
Score = 0, d	5.7	4.9	0.17	0.01	0.03
Score = 1, d	0.8	1.1	0.11	0.02	0.17
Score = 2, d	0.3	0.7	0.10	0.01	0.03
Score = 3, d	0.2	0.2	0.05	0.50	0.79
Total days of nasal scores					
Score = 0	40.3	39.9	0.50	0.61	—
Score = 1	1.3	1.3	0.36	0.94	—
Score = 2	0.4	0.8	0.25	0.35	—
Score = 3	—	—	—	—	—
Nasal score, d/wk					
Score = 0	6.7	6.7	0.08	0.50	0.90
Score = 1	0.2	0.2	0.05	0.94	0.94
Score = 2	0.1	0.1	0.04	0.29	0.87
Score = 3	—	—	—	—	—
Total days of eye/ear scores					
Score = 0	41.9	41.9	0.10	0.97	—
Score = 1	0.1	0.1	0.07	0.54	—
Score = 2	—	—	—	—	—
Score = 3	—	—	—	—	—
Eye/ear score, d/wk					
Score = 0	6.9	6.9	0.01	0.97	0.10
Score = 1	0.01	0.01	0.01	0.54	0.08
Score = 2	—	—	—	—	—
Score = 3	—	—	—	—	—

^1^Probability of *F*-test for treatment and treatment-by-week interaction.

Feeding calves 2× or 3× resulted in similar nasal and eye/ear scores (Table 8). Overall, calves were healthier when fed 2 times per day with a lower MR intake than calves fed 3 times per day. Knauer et al. (2018) stated that nutrient intake levels from milk is one management factor associated with pre weaning health and another was TSP. Although in this study, TSP concentrations were similar.

## Conclusions

The study objective was not achieved by feeding MR 3 times per day. This study demonstrated better growth performance, feed intake, feed conversions, frame measurement, and health performance when calves were fed 2 times per day due to greater CS intake during the weaning/transition period. The transition from a liquid to a solid feed should be as gradual as possible to enhance animal growth and welfare with later weaning. This study should be repeated by eliminating 1 feeding per week to facilitate a smoother/gradual transition to weaning. But, the current study also confirms that if calves are consuming sufficient CS that they can be weaned without a postweaning growth performance slump.

## References

[CIT0001] ADSA-ASAS-PSA. 2020. Guide for the care and use of agricultural animals in research and teaching. 4th ed. American Dairy Science Association, American Society of Animal Science and Poultry Science Association.

[CIT0002] AOAC. 2019. Official methods of analysis. 21st ed. Rockville (MD): AOAC International.

[CIT0003] Bach, A., L. Domingo, C. Montoro, and M. Terré. 2013a. Short communication: insulin responsiveness is affected by the level of milk replacer offered to young calves. J. Dairy Sci. 96:4634–4637. doi:10.3168/jds.2012-6196 23660138

[CIT0004] Bach, A., M. Terré, and A. Pinto. 2013b. Performance and health responses of dairy calves offered different milk replacer allowances. J. Dairy Sci. 96:7790–7797. doi:10.3168/jds.2013-6909 24119797

[CIT0005] Bai, Y., T. Liu, K. Hultquist, J. Wu, and D. P. Casper. 2020. Feeding an amino acid formulated milk replacer for Holstein calves. J. Anim. Sci. 98:ska099. doi:10.1093/jas/skaa099 PMC7323258 32266373

[CIT0007] Berends, H., H. van Laar, L. N. Leal, W. J. J. Gerrits, and J. Martín-Tereso. 2020. Effects of exchanging lactose for fat in milk replacer on ad libitum feed intake and growth performance in dairy calves. J. Dairy Sci. 103:4275–4287. doi:10.3168/jds.2019-17382 32113757

[CIT0057] Borderas, T. F., A. M. B. De passille, and J. Rushen. 2009. Feeding behavior of calves fed small or large amounts of milk. *J. Dairy Sci*. 92:2843–2852. doi:10.3168/.jds.2008-1886 19448018

[CIT0008] Casper, D. P., K. M. Hultquist, and I. P. Acharya. 2021. *Lactobacillus plantarum* GB LP-1 as a direct-fed microbial for neonatal calves. J. Dairy Sci. 104:5557–5568. doi:10.3168/jds.2020-19438. 33663862

[CIT0009] Chapman, C. E., P. S. Erickson, J. D. Quigley, T. M. Hill, H. G. Bateman, F. Suarez-Mena, and R. L. Schlotterbeck. 2016. Effect of milk replacer program on calf performance and digestion of nutrients with age of the dairy calf. J. Dairy Sci. 99:2740–2747. doi:10.3168/jds.2015-10372. 26805983

[CIT0010] Cowles, K. E., R. A. White, N. L. Whitehouse, and P. S. Erickson. 2006. Growth characteristics of calves fed an intensified milk replacer regimen with additional lactoferrin. J. Dairy Sci. 89:4835–4845. doi:10.3168/jds.S0022-0302(06)72532-2 17106114

[CIT0011] Davis, C. L., and J. K. Drackley. 1998. The development, nutrition, and management of the young calf. Ames (IA): Iowa State University Press.

[CIT0012] de Passillé Vieira, A. D. P., V. Guesdon, A. M. de Passille, M. A. G. von Keyserlingk, and D. M. Weary. 2008. Behavioural indicators of hunger in dairy calves. Appl. Anim. Behav. Sci. 109:180–189. doi: 10.1016/j.applanim.2007.03.006

[CIT0013] DuBois, M., K. A. Gilles, J. K. Hamilton, P. A. Rebers, and F. Smith. 1956. Colorimetric method for determination of sugars and related substances. Analytic Chem. 28:350–356. doi:10.1021/ac6011a017

[CIT0014] Eckert, E., H. E. Brown, K. E. Leslie, T. J. DeVries, and M. A. Steele. 2015. Weaning age affects growth, feed intake, gastrointestinal development, and behavior in Holstein calves fed an elevated plane of nutrition during the preweaning stage. J. Dairy Sci. 98:6315–6326. doi:10.3168/jds.2014-9062 26142851

[CIT0015] Erickson, P. S., J. L. Anderson, K. F. Kalscheur, G. J. Lascano, M. S. Akins, and A. J. Heinrichs. 2020. Symposium review: strategies to improve the efficiency and profitability of heifer raising. J. Dairy Sci. 103:5700–5708. doi:10.3168/jds.2019-17419 32147255

[CIT0016] Fischer, A. J., C. Villot, J. K. van Niekerk, T. T. Yohe, D. L. Renaud, and M. A. Steele. 2019. Invited Review: nutritional regulation of gut function in dairy calves: from colostrum to weaning. Appl. Anim. Sci. 35:498–510. doi:10.15232/aas.2019-01887

[CIT0017] Grice, K. D., K. M. Glosson, and J. K. Drackley. 2020. Effects of feeding frequency and protein source in milk replacer for Holstein calves. J. Dairy Sci. 103:10048–10059. doi:10.3168/jds.2020-19041 32952027

[CIT0018] Guindon, N. E., N. T. Antaya, R. G. Cabral, N. L. Whitehouse, T. J. Earleywine, and P. S. Erickson. 2015. Effects of human visitation on calf growth and performance of calves fed different milk replacer feeding levels. J. Dairy Sci. 98:8952–8961. doi:10.3168/jds.2015-9759 26476943 PMC7094226

[CIT0020] Hall, M. B. 2009. Determination of starch, including maltooligosaccharides, in animal feeds: comparison of methods and a method recommended for AOAC collaborative study. J. AOAC Int. 92:42–49. doi:10.1093/jaoac/92.1.42 19382561

[CIT0021] Heinrichs, A. J., G. I. Zanton, G. J. Lascano, and C. M. Jones. 2017. A 100-year review: a century of dairy heifer research. J. Dairy Sci. 100:10173–10188. doi:10.3168/jds.2017-12998 29153161

[CIT0022] Hill, T. M., J. D. Quigley, H. G. Bateman, F. X. Suarez-Mena, T. S. Dennis, and R. Schlotterbeck. 2016. Effect of milk replacer program on calf performance and digestion of nutrients in dairy calves to 4 months of age. J. Dairy Sci. 99:8103–8110. doi:10.3168/jds.2016-11239 27497902

[CIT0056] Jasper, J., and D. M. Weary. 2002. Effects of ad libitum milk intake on dairy calves. *J. Dairy Sci*. 85:3054–3058. doi:10.3168/jds.S0022-0302(02)74391-9 12487471

[CIT0023] Jaeger, B. M., D. Ziegler, D. Schimek, B. Ziegler, H. Chester-Jones, and D. P. Casper. 2020a. Dairy calf growth performance when fed a modified accelerated milk replacer program. Appl. Anim. Sci. 36:352–366. doi:10.15232/aas.2019-01878

[CIT0024] Jaeger, B. M., D. Ziegler, D. Schimek, B. Ziegler, H. Chester-Jones, and D. P. Casper. 2020b. Growth performance of newborn dairy calves fed a milk replacer containing 24% crude protein and 20% fat fed at different feeding rates. Appl. Anim. Sci. 36:208–218. doi:10.15232/aas.2019-01867

[CIT0025] Jensen, M. B., A. Jensen, and M. Vestergaard. 2020. The effect of milk feeding strategy and restriction of meal patterning on behavior, solid feed intake, and growth performance of male dairy calves fed via computer-controlled milk feeders. J. Dairy Sci. 103:8494–8506. doi:10.3168/jds.2020-18166 32684463

[CIT0055] Kehoe, S. I., C. D. Dechow, and A. J. Heinrichs. 2007. Effects of weaning age and milk feeding frequency on dairy calf growth, health, and rumen parameters. *Livestock Sci*. 110:267–272. doi:10.1016/j.livsci.2006.11.007

[CIT0027] Kesser, J., M. Korst, C. Koch, F. Romberg, J. Rehage, U. Müller, M. Schmicke, K. Eder, H. M. Hammon, H. Sadri, et al. 2017. Different milk feeding intensities during the first 4 weeks of rearing dairy calves: part 2: effects on the metabolic and endocrine status during calfhood and around the first lactation. J. Dairy Sci. 100:3109–3125. doi:10.3168/jds.2016-11595 28131581

[CIT0028] Khan, M. A., A. Bach, D. M. Weary, and M. A. G. von Keyserlingk. 2016. Invited review: transitioning from milk to solid feed in dairy heifers. J. Dairy Sci. 99:885–902. doi:10.3168/jds.2015-9975. 26709160

[CIT0029] Khan, M. A., H. J. Lee, W. S. Lee, H. S. Kim, K. S. Ki, T. Y. Hur, G. H. Suh, S. J. Kang, and Y. J. Choi. 2007. Structural growth, rumen development, and metabolic and immune responses of Holstein male calves fed milk through step-down and conventional methods. J. Dairy Sci. 90:3376–3387. doi:10.3168/jds.2007-0104 17582123

[CIT0030] Khan, M. A., D. M. Weary, and M. A. G. von Keyserlingk. 2011. Invited review: effects of milk ration on solid feed intake, weaning, and performance in dairy heifers. J. Dairy Sci. 94:1071–1081. doi:10.3168/jds.2010-3733 21338773

[CIT0031] Klopp, R. N., F. X. Suarez-Mena, T. S. Dennis, T. M. Hill, R. L. Schlotterbeck, and G. J. Lascano. 2019. Effects of feeding different amounts of milk replacer on growth performance and nutrient digestibility in Holstein calves to 2 months of age using different weaning strategies. J. Dairy Sci. 102:11040–11050. doi:10.3168/jds.2019-17153 31563311

[CIT0032] Klopp, R. N., F. X. Suarez-Mena, T. S. Dennis, T. M. Hill, R. L. Schlotterbeck, and G. J. Lascano. 2020. Postweaning response on growth and nutrient digestion to using different weaning strategies when feeding moderate and high amounts of milk replacer to Holstein calves. J. Dairy Sci. 103:8143–8150. doi:10.3168/jds.2019-17704 32684473

[CIT0033] Kmicikewycz, A. D., D. N. L. da Silva, J. G. Linn, and N. B. Litherland. 2013. Effects of milk replacer program fed 2 or 4 times daily on nutrient intake and calf growth. J. Dairy Sci. 96:1125–1134. doi:10.3168/jds.2012-5738. 23219113

[CIT0034] Knauer, W. A., S. M. Godden, S. M. McGuirk, and J. Sorg. 2018. Randomized clinical trial of the effect of a fixed or increasing milk allowance in the first 2 weeks of life on health and performance of dairy calves. J. Dairy Sci. 101:8100–8109. doi:10.3168/jds.2017-14309 29908803

[CIT0035] Korst, M., C. Koch, J. Kesser, U. Müller, F. Romberg, J. Rehage, K. Eder, and H. Sauerwein. 2017. Different milk feeding intensities during the first 4 weeks of rearing in dairy calves: Part 1: effects on performance and production from birth over the first lactation. J. Dairy Sci. 100:3096–3108. doi:10.3168/jds.2016-11594 28131579

[CIT0036] Kovács, L., F. L. Kézér, P. Póti, N. Boros, and K. Nagy. 2020. Short communication: upper critical temperature-humidity index for dairy calves based on physiological stress variables. J. Dairy Sci. 103:2707–2710. doi:10.3168/jds.2019-17459 31864752

[CIT0037] Krishnamoorthy, U., T. V. Muscato, C. J. Sniffen, and P. J. Van Soest. 1982. Nitrogen fractions in selected feedstuffs. J. Dairy Sci. 65:217–225. doi:10.3168/jds.s0022-0302(82)82180-2

[CIT0038] Liu, T., K. Hultquist, K. Froehlich, and D. P. Casper. 2020. Feeding an amino acid–formulated milk replacer for Holstein calves during 2 time periods. J. Dairy Sci. 103:10108–10121. doi:10.3168/jds.2020-18529 32921466

[CIT0039] McGuirk, S. 2013. Calf health scoring chart. Calf Health Scoring Chart. University of Wisconsin, School of Veterinary Medicine. [accessed September 01, 2015]. https://www.vetmed.wisc.edu/dms/fapm/ fapmtools/8calf/calf_health_scoring_chart.pdf

[CIT0040] Mirzaei, M., N. Dadkhah, B. Baghbanzadeh-Nobari, A. Agha-Tehrani, M. Eshraghi, M. Imani, R. Shiasi-Sardoabi, and M. H. Ghaffari. 2018. Effects of preweaning total plane of milk intake and weaning age on intake, growth performance, and blood metabolites of dairy calves. J. Dairy Sci. 101:4212–4220. doi:10.3168/jds.2017-13766 29477525

[CIT0041] Mirzaei, M., M. Khorvash, G. R. Ghorbani, M. Kazemi-Bonchenari, and M. H. Ghaffari. 2017. Growth performance, feeding behavior, and selected blood metabolites of Holstein dairy calves fed restricted amounts of milk: no interactions between sources of finely ground grain and forage provision. J. Dairy Sci. 100:1086–1094. doi:10.3168/jds.2016-11592 28012617

[CIT0042] National Academies of Sciences, Engineering, and Medicine. 2021. Nutrient requirements of dairy cattle. 8th rev. ed. Washington, DC. The National Academies Press. doi:10.17226/25806 38386771

[CIT0043] NRC. 2001. Nutrient requirements of dairy cattle. 7th rev. ed. Washington, DC: Natl. Acad. Press.

[CIT0044] Omidi-Mirzaei, H., M. Khorvash, G. R. Ghorbani, B. Moshiri, M. Mirzaei, A. Pezeshki, and M. H. Ghaffari. 2015. Effects of the step-up/step-down and step-down milk feeding procedures on the performance, structural growth, and blood metabolites of Holstein dairy calves. J. Dairy Sci. 98:7975–7981. doi:10.3168/jds.2014-9260 26319763

[CIT0045] Quigley, J. 2007. Calculating ME in milk and milk replacers. Calf Note #122. [accessed April 06, 2022]. https://calfnotes.com/pdffiles/CN122.pdf

[CIT0046] Roland, L., M. Drillich, D. Klein-Jöbstl, and M. Iwersen. 2016. Invited review: Influence of climatic conditions on the development, performance, and health of calves. J. Dairy Sci. 99:2438–2452. doi:10.3168/jds.2015-9901 26874416

[CIT0047] Schingoethe, D. J., D. P. Casper, J. K. Drackley, and F. C. Ludens. 1986. Increased solids intake and feeding frequency for calves in hutches during cold weather. J. Dairy Sci. 69:1063–1069. doi:10.3168/jds.s0022-0302(86)80502-1 3722528

[CIT0048] Steele, R. G., and J. H. Torrie. 1980. Principles and procedures of statistics: a biometrical approach. New York: McGraw-Hill.

[CIT0049] Van Amburgh, M. E., F. Soberon, M. J. Meyer, and R. A. Molano. 2019. Integration of postweaning nutrient requirements and supply with composition of growth and mammary development in modern dairy heifers. J. Dairy Sci. 102:3692–3705. doi:10.3168/jds.2018-15270 30660424

[CIT0050] van Niekerk, J. K., A. J. Fischer-Tlustos, J. N. Wilms, K. S. Hare, A. C. Welboren, A. J. Lopez, T. T. Yohe, L. R. Cangiano, L. N. Leal, and M. A. Steele. 2021. ADSA foundation scholar award: new frontiers in calf and heifer nutrition—from conception to puberty. J. Dairy Sci. 104:8341–8362. doi:10.3168/jds.2020-20004 34053756

[CIT0051] Vitali, A., M. Segnalini, L. Bertocchi, U. Bernabucci, A. Nardone, and N. Lacetera. 2009. Seasonal pattern of mortality and relationships between mortality and temperature-humidity index in dairy cows. J. Dairy Sci. 92:3781–3790. doi:10.3168/jds.2009-2127 19620660

[CIT0052] Wang, J., J. Li, F. Wang, J. Xiao, Y. Wang, H. Yang, S. Li, and Z. Cao. 2020. Heat stress on calves and heifers: a review. J. Anim. Sci. Biotechnol. 11:1–79. doi:10.1186/s40104-020-00485-8 32789013 PMC7416401

[CIT0054] Welboren, A. C., B. Hatew, O. López-Campos, J. P. Cant, L. N. Leal, J. Martín-Tereso, and M. A. Steele. 2021. Effects of energy source in milk replacer on glucose metabolism of neonatal dairy calves. J. Dairy Sci. 104:5009–5020. doi:10.3168/jds.2020-19405 33612241

[CIT0053] Welboren, A. C., L. N. Leal, M. A. Steele, M. A. Khan, and J. Martín-Tereso. 2019. Performance of ad libitum fed dairy calves weaned using fixed and individual methods. Animal. 13:1891–1898. doi:10.1017/S1751731119000181 30789110

